# Genome-Wide Analysis of the RAV Family in Soybean and Functional Identification of *GmRAV-03* Involvement in Salt and Drought Stresses and Exogenous ABA Treatment

**DOI:** 10.3389/fpls.2017.00905

**Published:** 2017-06-06

**Authors:** Shu-Ping Zhao, Zhao-Shi Xu, Wei-Jun Zheng, Wan Zhao, Yan-Xia Wang, Tai-Fei Yu, Ming Chen, Yong-Bin Zhou, Dong-Hong Min, You-Zhi Ma, Shou-Cheng Chai, Xiao-Hong Zhang

**Affiliations:** ^1^College of Agronomy/College of Life Sciences, Northwest A&F University/State Key Laboratory of Crop Stress Biology for Arid AreasYangling, China; ^2^Institute of Crop Sciences, Chinese Academy of Agricultural Sciences/National Key Facility for Crop Gene Resources and Genetic Improvement, Key Laboratory of Biology and Genetic Improvement of Triticeae Crops, Ministry of AgricultureBeijing, China; ^3^Shijiazhuang Academy of Agricultural and Forestry Sciences, Research Center of Wheat Engineering Technology of HebeiShijiazhuang, China

**Keywords:** ABA, abiotic stress response, RAV family, salt tolerance, soybean

## Abstract

Transcription factors play vital roles in plant growth and in plant responses to abiotic stresses. The RAV transcription factors contain a B3 DNA binding domain and/or an APETALA2 (AP2) DNA binding domain. Although genome-wide analyses of *RAV* family genes have been performed in several species, little is known about the family in soybean (*Glycine max* L.). In this study, a total of 13 *RAV* genes, named as GmRAVs, were identified in the soybean genome. We predicted and analyzed the amino acid compositions, phylogenetic relationships, and folding states of conserved domain sequences of soybean RAV transcription factors. These soybean RAV transcription factors were phylogenetically clustered into three classes based on their amino acid sequences. Subcellular localization analysis revealed that the soybean RAV proteins were located in the nucleus. The expression patterns of 13 *RAV* genes were analyzed by quantitative real-time PCR. Under drought stresses, the *RAV* genes expressed diversely, up- or down-regulated. Following NaCl treatments, all RAV genes were down-regulated excepting *GmRAV-03* which was up-regulated. Under abscisic acid (ABA) treatment, the expression of all of the soybean *RAV* genes increased dramatically. These results suggested that the soybean *RAV* genes may be involved in diverse signaling pathways and may be responsive to abiotic stresses and exogenous ABA. Further analysis indicated that *GmRAV-03* could increase the transgenic lines resistance to high salt and drought and result in the transgenic plants insensitive to exogenous ABA. This present study provides valuable information for understanding the classification and putative functions of the RAV transcription factors in soybean.

## Introduction

Soybean (*Glycine max* L.) is an economically important crop that is used in human diets, animal feeds and for biodiesel production. However, soybean growth and productivity are greatly affected by environmental stresses such as drought and high soil salinity (Kang et al., 2009; Kidokoro et al., 2015). Plants can resist abiotic stresses by regulating the expression of a variety of genes. Under stress conditions, transcriptome changes are the earliest responses before biosynthesis, modification, and interaction of related expression proteins in plants (Yamaguchi-Shinozaki and Shinozaki, 2006). The expression of genes encoding transcription factors are induced or repressed and these function to further regulate plant responses to environmental stresses (Hao and O’Shea, 2012). Many transcription factors involved in plant stress-resistance pathways have been identified, among which the ‘related to ABI3/VP1 (RAV)’ family of transcription factors are one of them. The RAV family is part of the B3 superfamily, which also contains the ARF, LAV, and REM families. The B3 superfamily is defined by the B3 domain, which consists of about 110 amino acids (Swaminathan et al., 2008). The RAV family proteins contain a B3 domain and/or an AP2 domain. Thus, the RAV family members could reasonably be classified as members of either the B3 superfamily or the AP2/EREBP family (Matías-Hernández et al., 2014). The AP2 domain as a DNA-binding domain was first identified in the *Arabidopsis* (*Arabidopsis thaliana*) AP2 protein (Jofuku et al., 1994). The B3 domain is a DNA-binding domain (Finkelstein et al., 1998) that was initially named due to its position in the third basic domain of the maize gene *VIVIPAROUS1* (*VP1*) (McCarty et al., 1991). Unlike the first and second basic domains (B1 and B2) which are specific to the VP1-like proteins, the B3 domain is widespread in plant genomes (Suzuki et al., 1997). B3 domains consist of seven β-barrels and two short α-helices (Yamasaki et al., 2004; Waltner et al., 2005). *Arabidopsis* RAV1 and RAV2, which each contain both a B3 domain and an AP2 domain, were the first identified members of the RAV family (Kagaya et al., 1999).

The members of RAV family play important roles in plant physiological processes, such as leaf senescence, flowering development, organ growth, and hormone signaling. *Arabidopsis RAV1* (Hu et al., 2004) and soybean *GmRAV* negatively regulate SD-mediated flowering and hypocotyl elongation and overexpression of *GmRAV* in SDs may inhibite the growth of soybean leaf, root, and stem suggesting they act in prominent roles in controlling plant growth (Zhao et al., 2008; Lu et al., 2014). It was reported that the RAV1 transcription factor play important roles in positively regulating leaf senescence in *Arabidopsis* (Woo et al., 2010). The *Arabidopsis NGATHA* genes (*NGA1*-*NGA4*) which belong to RAV family play important roles in leaf and flower development. *nga1/nga2/nga3/nga4* quadruple mutant exhibits leaf shape defects and several flower defects (Alvarez et al., 2006; Lee et al., 2015). Matías-Hernández et al. (2014) summarized the functions of RAV genes in controlling flowering in different pathways. For instance, *TEM1* and *TEM2* which also belong to RAV family can delay flowering by repressing the production of FLOWERINGLOCUS T (FT) and gibberellins (Matías-Hernández et al., 2014). And *Arabidopsis* overexpressing *BrNGA1* displayed markedly reduced organ growth compared with the WT (Kwon et al., 2009).

It was reported that RAV transcription factors function in the regulation of plant responses to plant pathogens. Tomato RAV transcription factors may act as intermediate transcription factors that somehow connect *AtCBF1* and pathogenesis-related genes, conferring enhanced tolerance to bacterial wilt (Li et al., 2011). Overexpression of the pepper *CARAV1* gene in transgenic *Arabidopsis* plants was demonstrated to induce the expression of some pathogenesis-related genes, and to confer resistance to *Pseudomonas syringae* pv. *tomato* DC3000 to confer tolerance to osmotic stress (Lee and Hwang, 2006; Sohn et al., 2006). Therefore, it is clear that the RAV family members mediate plant growth and developmental process and that these proteins are responsive to diverse hormone/pathogenic bacteria stimuli.

Meanwhile, the expression levels of several RAV family members were affected by various plant hormones. RAV proteins are known to be involved in ethylene and brassinosteroid responses (Alonso et al., 2003; Hu et al., 2004). In *Arabidopsis TEMPRANILLO* (TEM) genes repressed the expression of GA4 biosynthetic genes, resulting in retarded-growth phenotypes of transgenic plants (Osnato et al., 2012). *RAV1* (Hu et al., 2004) and AT3G25730 (*RAV1L)* in *Arabidopsis* were down-regulated by 24-epibrassinolide (epiBL) and ABA, respectively. And the *RAV1*-overexpressing transgenic *Arabidopsis* were insensitive to ABA (Fu et al., 2014). ABA as the important one of plant hormones plays vital roles in controlling plant abiotic stress responses. The production of ABA can be triggered by drought and high salinity, and ABA activates some drought-inducible genes (Yang et al., 2016).

Nevertheless, to date, little has been reported regarding the function(s) RAV proteins in plant responses to abiotic stress. In cotton (*Gossypium hirsutum*), overexpression AtRAV1/2 could improve the drought resistance of cotton (Matías-Hernández et al., 2014). The pepper *CARAV1* which was induced by abiotic stresses functioned as a transcriptional activator triggering tolerance to osmotic stresses (Sohn et al., 2006). Lee et al. (2010) also reported that the overexpression *Arabidopsis* of pepper *CARAV1* increased tolerance to high salinity stresses. These results indicate that RAV proteins may play roles in plant responses to various abiotic stresses. Given the potential importance of RAV genes in plant responses to abiotic stress environments, we carried out a genome-wide analysis of the soybean *RAV* gene family and investigated the potential functions of *RAV* genes in plant responses to various experimental stimuli.

## Materials and Methods

### The Searches for *RAV* Genes in the Soybean, *Arabidopsis*, and Rice Genomes Database

Data for the whole genome sequences and predicted RAV protein sequences of soybean, *Arabidopsis* and rice were obtained from the JGI Glyma1.0 annotation, TAIR and TIGR, respectively. The gene chip data for soybean were downloaded from SoyBase^1^.

### Identification and Genomic Locations of Soybean RAVs

To concatenate the probable RAV family members in soybean, all publicly known *Arabidopsis RAV* genes were used as protein queries using analysis tools available at the Phytozome website^2^, and candidate genes were identified based on BLASTP searching, using distance score value ≥100 and an e-value ≤ 1e^-10^ (Goodstein et al., 2012). Next, the Pfam database was used to determine if each candidate RAV sequence was a member of the RAV family. To exclude repeated sequences, all candidate RAV sequences were aligned using ClustalX and checked manually. A total of 13 soybean RAVs were obtained after manually filtering out repeated sequences. All non-redundant RAVs were mapped to eight soybean chromosomes using MapDraw software (Liu and Meng, 2003).

### Gene Structure and *cis*-Acting Element Analysis

An exon–intron substructure map was produced for each of the genomic loci of the 13 *RAV* genes using Tools Online GSDS (Guo et al., 2007). Soybean *RAV* promoters were evaluated using Promoter 2.0 Prediction Server^3^ (Knudsen, 1999). *Cis*-acting elements were analyzed using the plant *cis-*acting element database PLACE 26.0 which has closed in 2017 (Higo et al., 1999) and PlantCARE which is still operating (Lescot et al., 2002).

### Alignment and Phylogenetic Analysis of RAVs Sequences

Multiple alignments of the predicted amino acid sequences were performed using ClustalX and were manually corrected. For generating the phylogenetic tree, we used ClustalX (1.83) and the neighbor-joining (NJ) algorithm in MEGA7.0 Bootstrap analysis with 1,000 replicates was used to evaluate the significance of nodes. Representations of the calculated trees were constructed using TreeView. Bootstrapping was performed 500 times to obtain support values for each branch.

### Microarray Analysis of *RAV* Gene Expression Patterns

An analysis was conducted using the Affimetrix soybean gene chip downloaded in SoyBase^1^, the analysis was carried out which included the 13 soybean RAVs in the different tissues and development stages. And the heatmap was generated by the software HemI.

### Plant Materials and Stress Treatments

Soybean seeds (*Tiefeng 8*) were germinated in vermiculite in a light chamber at 25°C. 14-day-old seedlings were subjected to various abiotic stresses, including drought, salinity, and treatment with exogenous ABA. For the drought stress, soybean seedlings were removed from the soil, and dehydrated for 0, 2, 4, 8, 12, and 24 h. For the other treatments, seedlings were transferred to solutions containing 200 mM NaCl and 200 μM ABA, respectively. Seedlings were sampled at 0, 2, 4, 8, 12, and 24 h after commencing the various treatments. Harvested seedlings were dropped immediately into liquid nitrogen and stored at -80°C until RNA extraction.

### Subcellular Localization Analysis in *Arabidopsis* Protoplasts

Thirteen expression vectors with GFP tags were constructed for subcellular localization analysis, as described previously (Li et al., 2012a). Under control of the CaMV35S promoter, the amplified coding regions were fused to the N terminal region of GFP (Supplementary Table S1). The subcellular localization of the GFP expression in *Arabidopsis* protoplasts was monitored by confocal microscopy 16 h after polyethylene glycol mediated transformation, as described previously (Yoo et al., 2007).

### Reverse Transcription-PCR (RT-PCR) and Quantitative Real-Time PCR (qRT-PCR)

Based on the manufacturer’s instructions of RNeasy Plant Mini Kit (Qiagen), the total RNA was isolated from whole plants. cDNA synthesis and RT-PCR were conducted as previously described (Xu et al., 2007). Then the expression patterns were analyzed with an ABI Prism 7300 sequence detection system (Applied Biosystems) as previously described (Li et al., 2012b, 2014). The qRT-PCR primers of soybean *RAV* genes were designed by Primer Premier 5.0 software (Supplementary Table S1).

### Tolerance Assays under Stress Conditions

The *GmRAV-03* gene, which is induced by the drought, salt, and exogenous ABA, was selected to confirm gene functions. Fragment of *GmRAV-03* was ligated into the pCAMBIA1302 vector under control of the CaMV 35S promoter using the primers in Supplementary Table S1. The expression vector pCAMBIA1302::GmRAV-03 was transformed into the *Agrobacterium tumefaciens* (GV3101), followed by *Arabidopsis* transformation using the floral dipping method (Clough and Bent, 1998; Fu et al., 2014). Transgenic *Arabidopsis* lines were selected using MS medium (Supplementary Table S2) containing 30 mg/L hygromycin. Further experiments were performed with homozygous lines in T3 generation. And based on the expression levels of various lines containing four transgenic lines and wild line, we selected OE-2 and OE-3 for further experiments (**Supplementary Figure S1**).

For the germination assays, the seeds of Col-0 and transgenic lines were surface sterilized and kept at 4°C for 3 days in the dark before germination. About 100 seeds of every genotype were sown on the same plate containing 1/2 MS medium with or without ABA and NaCl and were in a growth room kept at 22°C, 40 μmol m^-2^ sec^-1^ light with 16 h of light and 8 h of darkness as described previously (Feng et al., 2014). Each day germinated seeds with protruded radicles were counted (Feng et al., 2014).

To assess the phenotype under stress conditions, seedlings grown on 1/2 medium for 4 days were transferred to 1/2 MS medium with or without ABA, PEG, and NaCl, and kept at 22°C, 40 μmol m^-2^ sec^-1^ light with 16 h of light and 8 h of darkness as described previously (Feng et al., 2014). For phenotypic evaluations, we used Expression 11000xl to sweep the seedling root and used the software called WinRHIZO to measure the seedling root length, and root surface at least 10 days after treatment with NaCl or ABA and 14 days after treatment with PEG6000. At least 27 seedlings were measured for each line and each treatment (Lin et al., 2013). All stress assays were performed at least three times and the representative data were shown in the figures.

Two-week-old transgenic *Arabidopsis* soil-grown plants were treated with 300 mM NaCl solution by flush flooding (to soak the soil for a short period), while the control group was watered (Das et al., 2016). After 10 days, the number of survival seedlings was counted. To measure the chlorophyll, 200 mg leaf tissues were harvested at 7 days post-treatment. Chlorophyll was quantified photometrically (Porra et al., 1989). Two-week-old transgenic *Arabidopsis* soil-grown plants were treated with 200 μM exogenous ABA by spray on the leaves, while the control group was sprayed with water. After 2 weeks, plant height was measured by rule. 10 days transgenic *Arabidopsis* and wild *Arabidopsis* were transferred to barrels filled with a mixture of soil and sand (1:1). And water supply was withdrawn. After severe drought stress, water was added for recovery, and survival performance was photographed and recorded (Guo et al., 2016; Lu et al., 2016). At least 45 seedlings were measured for each line and each treatment. All stress assays were performed at least three times.

## Results

### Identification of RAV Transcription Factors in Soybean

Hundred and twenty seven putative soybean B3 sequences were acquired via BLASTP searches in the JGI Glyma1.0 annotation. After eliminating repeated sequences, 13 non-redundant soybean RAVs were putatively identified (**Table 1**). The polypeptide lengths of the predicted soybean RAVs varied widely, ranging from 288 to 582. The predicted isoelectric points of the proteins were also diverse (**Table 1**).

**Table 1:** Protein information for the soybean RAVs, including sequence ID, predicted protein sequence length, predicted molecular weight (MW), predicted isoelectric point (p*I*), and chromosome locations of the genes putatively encoding these proteins.

Number	Gene name	Gene ID number	Amino acid residues	MW (Da)	pI	B3 domain	AP2 domain	Chromosome	Location in chromosome
1	*GmRAV-01*	Glyma01G22260	384	41907.1	9.6	205~312	75~123	1	55.14
2	*GmRAV-02*	Glyma02G36090	344	39079.7	6.9	75~181		2	38.4
3	*GmRAV-03*	Glyma02G11060	401	43610.2	9.5	210~324	81~129	2	9.31
4	*GmRAV-04*	Glyma03G35700	288	32721.9	7.1	68~167		3	40.8
5	*GmRAV-05*	Glyma03G42301	420	47128.1	7.3	88~191		3	45.56
6	*GmRAV-06*	Glyma07G05381	491	55486.7	7.1	131~225		7	4.06
7	*GmRAV-07*	Glyma10G08871	337	38494	7	73~178		10	7.92
8	*GmRAV-08*	Glyma10G34760	351	38379.3	8	172~269	52~100	10	43.55
9	*GmRAV-09*	Glyma16G01951	582	65550.7	7.5	195~293		16	1.48
10	*GmRAV-10*	Glyma19G38340	299	34108.2	6.9	77~181		19	45.4
11	*GmRAV-11*	Glyma19G45090	413	45902.9	8.1	90~194		19	50.4
12	*GmRAV-12*	Glyma20G39140	320	36244.6	7.8	158~248	37~83	20	47.6
13	*GmRAV-13*	Glyma20G32730	362	39343.6	9.3	178~275	58~106	20	42.4

**FIGURE 1:**
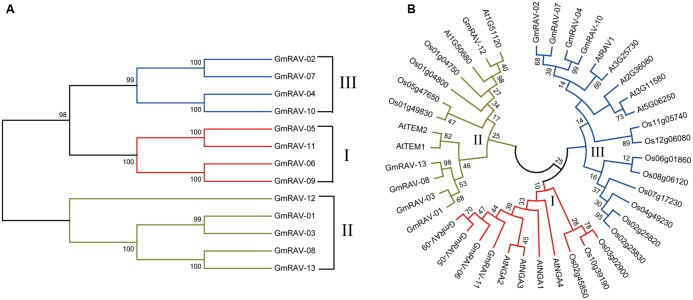
Phylogenetic relationships of RAV family proteins in soybean, *Arabidopsis*, and rice. The phylogenetic tree was produced using MEGA 7.0 software based on the comparison of amino acid sequences of the RAV proteins. The neighbor-joining method was used and the bootstrap replicates were set at 1000. **(A)** Neighbor-joining phylogenetic tree of the RAV family in soybean. **(B)** Phylogenetic relationship of the RAV families of Gm (*Glycine max*), At (*Arabidopsis thaliana*), and Os (*Oryza sativa*). Soybean RAVs were divided into three classes (I, II, III).

**FIGURE 2:**
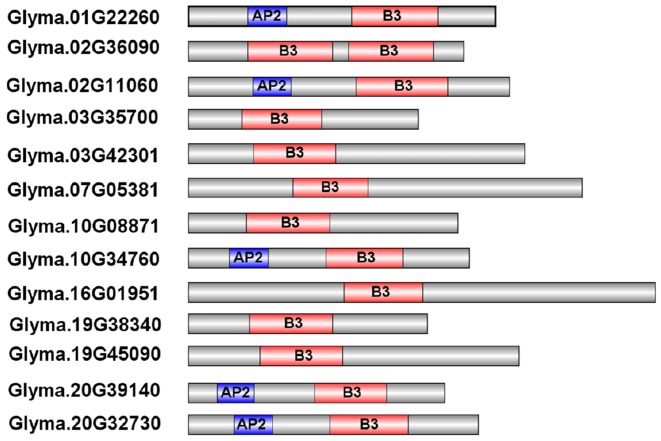
Predicted structures of the RAV proteins in soybean. The B3 domains and AP2 domains are indicated by the red and blue boxes, respectively.

To evaluate the phylogenetic relationships among the soybean RAVs, a phylogenetic analysis of the 13 soybean RAVs, 15 rice RAVs, and 13 *Arabidopsis* RAVs was undertaken based on a phylogenetic tree generated with a neighbor-joining method (**Figure 1**). The family was divided into three groups (**Figure 1**). 5 of the 13 genes soybean *RAV* genes, including *GmRAV-01*, *GmRAV-03*, *GmRAV-10*, *GmRAV-12*, and *GmRAV-13*, contain an AP2 domain in addition to the B3 domain (**Figure 2**). This result is very similar to the situation observed for the RAVs in *Arabidopsis*, where 6 of the 13 RAV proteins contain an AP2 domain (Swaminathan et al., 2008). The other members of the soybean and *Arabidopsis* RAV family contain only a B3 domain (**Figure 2**). And the major difference, when comparing soybean and *Arabidopsis*, is genomic locations of RAVs. In soybean, the number of RAV genes in each of chromosomes is almost consistent. The eight chromosomes contain 1∼2 RAV genes, respectively (**Supplementary Figure S2A**). However, in *Arabidopsis*, chromosome 1 carried six RAV genes, whereas only 1∼3 RAV genes was present in other four chromosomes, respectively (**Supplementary Figure S2B**).

**FIGURE 3:**
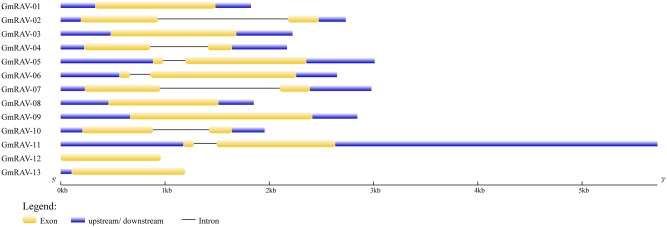
Intron–exon structures of the soybean *RAV* genes. The intron–exon structures were produced using the GSDS online tool. The exons, introns, and untranslated regions (UTRs) are indicated, respectively, by the white boxes, black lines, and gray lines.

### Gene Structure and *cis*-Acting Elements

Gene structure analysis revealed the existence of introns in the soybean *RAV* genes. In the soybean family, *RAV* genes containing no introns accounted for 46.15% of the total, and genes containing one intron accounted for the remaining 53.85%. We obtained the data of *cis*-elements from two different database PLACE which has closed and PlantCARE. *Cis*-element analysis in database PLACE demonstrated that every soybean *RAV* member carried two or more of each of the following in their promoters: E-BOX, GT-1, MYB, and MYC elements. In addition, 76.9% of the genes contained two or more ABREs. The soybean *RAV* promoters had no or only one low-temperature responsive element (LTRE) (**Table 2**). And in database PlantCARE, most members still contained abiotic related *cis*-elements, such as ABRE, MBS, HSE, and TC-rich repeats except the light related *cis*-element Box 4. Analyses of *cis*-elements in the promoters of the soybean RAV family contribute to conclude its predicted function.

**Table 2:** Distribution of abiotic stress-related *cis*-acting elements in soybean *RAV* gene promoters.

Data from PLACE						
**Gene**	**ABRE**	**E-BOX**	**GT-1**	**LTRE**	**MYB**	**MYC**
GmRAV-01	2	18	32	0	12	18
GmRAV-02	2	4	32	1	18	6
GmRAV-03	1	14	29	1	11	18
GmRAV-04	6	14	31	1	10	14
GmRAV-05	3	12	21	1	7	12
GmRAV-06	3	14	23	0	18	20
GmRAV-07	4	6	35	1	10	10
GmRAV-08	0	4	46	0	9	4
GmRAV-09	6	28	27	0	21	28
GmRAV-10	6	16	25	0	14	18
GmRAV-11	3	10	18	0	9	10
GmRAV-12	7	10	30	0	19	10
GmRAV-13	1	2	33	0	11	2

**Data from PlantCARE**						

**Gene**	**ABRE**	**ARE**	**HSE**	**MBS**	**TC-rich repeats**	**Box 4**

GmRAV-01	1	2	1	1	2	2
GmRAV-02	2	3	2	2	1	1
GmRAV-03	0	2	1	2	1	3
GmRAV-04	1	2	3	0	1	0
GmRAV-05	2	1	1	3	2	2
GmRAV-06	1	0	1	3	1	0
GmRAV-07	4	3	2	1	1	1
GmRAV-08	0	6	2	0	2	3
GmRAV-09	2	1	0	4	4	1
GmRAV-10	1	2	3	0	1	1
GmRAV-11	2	1	1	4	1	4
GmRAV-12	2	0	1	2	5	1
GmRAV-13	0	1	0	0	0	0

### The Predicted Structural Features of Conserved Domains in RAV Proteins in Soybean

**FIGURE 4:**
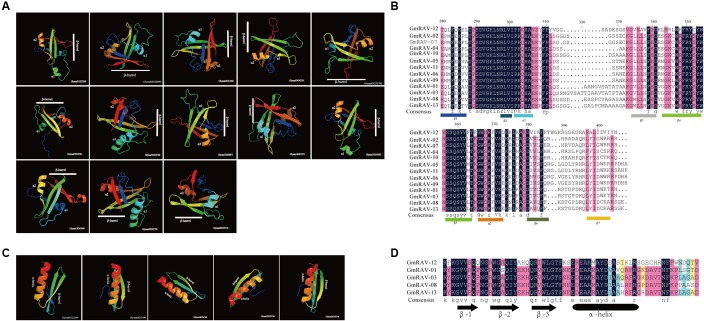
Predicted structure of conserved domains in RAV proteins in soybean. **(A)** Ribbon diagrams of the B3 domains of the soybean RAV family. **(B)** Alignment of the B3 domains of the soybean RAV family. **(C)** Ribbon diagrams of the AP2 domains of soybean RAV family. **(D)** Alignment of the AP2 domains of the soybean RAV family.

To explore the potential structural features of the conserved domains of RAV proteins of the soybean, multiple alignment analyses were performed using the amino acid sequences of the B3 and AP2 domains, respectively. All B3 domains in 13 RAV proteins have the same structural feature which is the presence of seven β-strands (β1–β7) that come together to form an open β-barrel. Two α-helices (α1, α2) are present between β-strands 2 and 3 and between β-strands 5 and 6. The α1 and α2 helices project from opposite ends of the β-barrel (**Figures 4A,B**).

AP2 domains in five RAV proteins are the presence of three β-barrels that parallel with one α-helix (**Figures 4C,D**). These observations are generally consistent with the previous reports about structural features of the B3 and AP2 domains of plants (Nakano et al., 2006; Swaminathan et al., 2008).

### Subcellular Localization of Soybean RAVs

**FIGURE 5:**
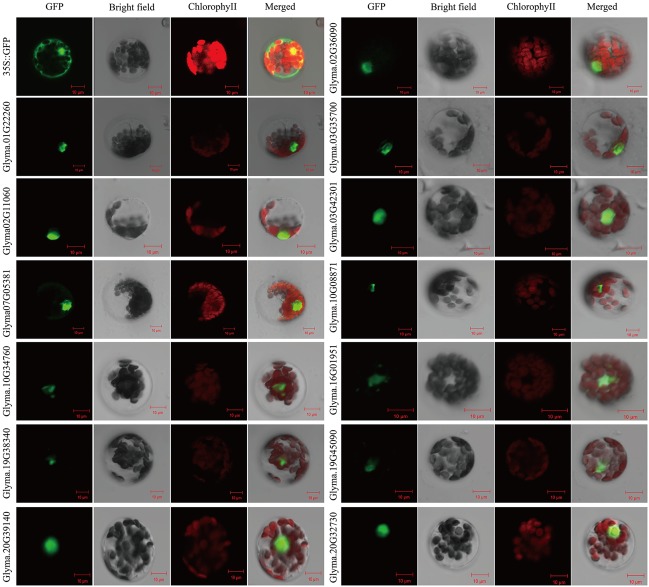
Subcellular localization of the soybean RAV proteins. Results were visualized with confocal microscopy 16 h after transformation. Scale bars = 10 μm.

To determine the subcellular localization of soybean RAV proteins, the full length cDNA sequences of 13 *RAV* genes were fused to the C-terminus of the hGFP reporter genes and subcloned into an expression vectors under the control of the CaMV35S promoter. Subcellular localization of GFP expression in soybean mesophyll protoplasts was observed after transformation. The empty 35S::GFP vector was transformed as the control. Soybean RAV fusion proteins were all localized in the cell nucleus (**Figure 5**), which suggests that they are nuclear proteins, likely functioning as transcription factors.

### Expression Patterns of Soybean *RAV* Genes

**FIGURE 6:**
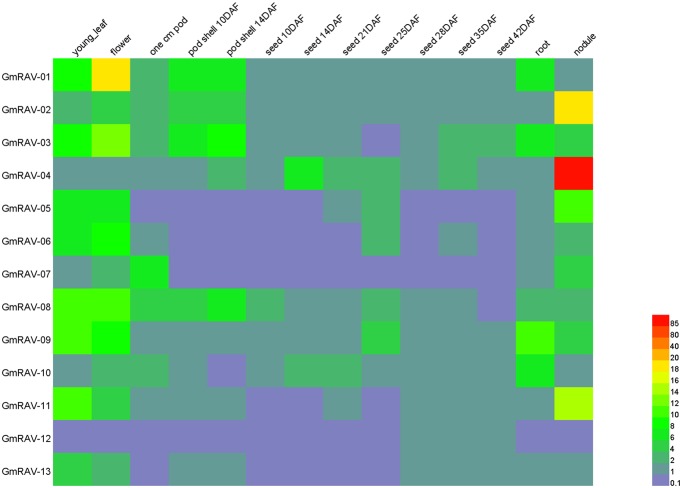
Expressions of soybean *RAV* genes. Differential expression analysis of *RAV* genes in soybean.

The expression pattern of a gene often has a close relationship with its function (Wan et al., 2014). ESTs and cDNA data, which are available in various databases are useful resources for analysis of gene expression (Adams et al., 1995). To examine the expression patterns in different soybean tissues and organs, an expression pattern heatmap of the soybean *RAV* genes was drawn based on data downloaded from the soybean genome database (**Figure 6** and Supplementary Table S3). Analysis of the expression of the 13 different *RAV* genes in 14 different tissues and organs and at different developmental stages revealed that a wide scope of differential expression patterns among the genes. For example, *GmRAV-12*, which was only expressed in flower; whereas, *GmRAV-01*, *GmRAV-03*, *GmRAV-05*, *GmRAV-06*, *GmRAV-08*, *GmRAV-07*, *GmRAV-09*, *GmRAV-10*, and *GmRAV-11* were expressed in flower, young leaf, pod, seed, root, and nodule. Expression levels of different genes in same tissue were also disparate. For example, in flower, *GmRAV-01* expression levels were 19 times as much as *GmRAV-04* expression levels. Nevertheless, in nodule, *GmRAV-04* expression levels were 89 times of *GmRAV-01* expression levels. Even the same gene in different tissues, its expression levels were also varied. *GmRAV-02* transcripts reached maximum levels in nodule, but it was not expressed in root and seed (**Figure 6** and Supplementary Table S3).

### qRT-PCR Analyses of Soybean RAV Genes

**FIGURE 7:**
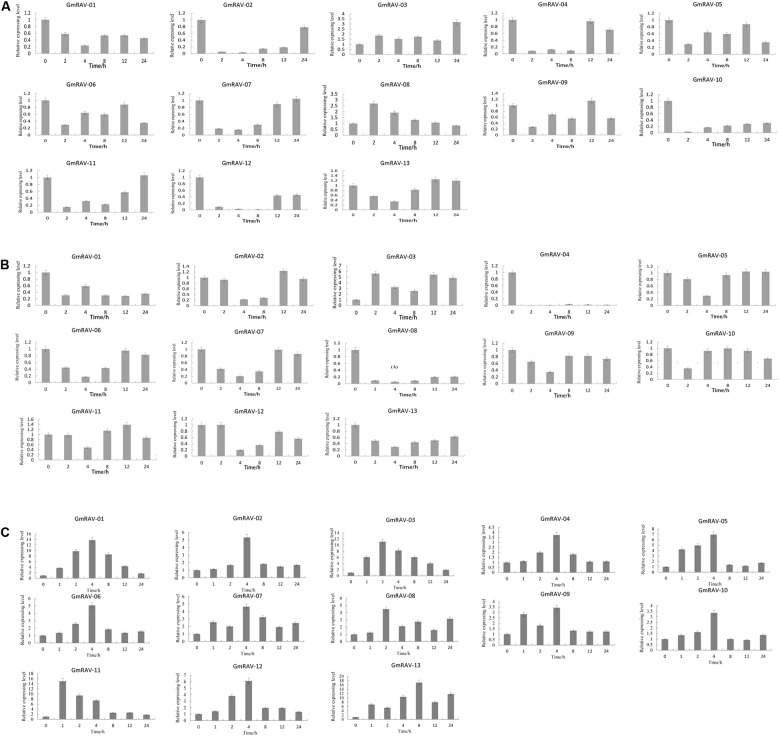
Relative expression of soybean *RAV* genes in plants treated with drought, NaCl, and exogenous application of ABA. qRT-PCR analyses of plants treated with drought **(A)**, NaCl **(B),** and ABA **(C)**.

Abiotic stresses and phytohormones are known to affect a great number of physiological processes. To investigate the potential functions of the soybean *RAV* genes in response to different stimuli, the expression patterns of these genes in plants that had been treated with ABA or grown in drought and salt stress conditions were analyzed by qRT-PCR (**Figure 7**). Under drought stress, two genes were obviously up-regulated (>2-fold); others were down-regulated slightly or obviously (**Figure 7A**). Following salt treatment, all genes, excepting *GmRAV-03* which was up-regulated, were down-regulated (**Figure 7B**). Exogenous ABA also affected the transcription of the soybean *RAV* gene. Under ABA treatment, the expression of all of the soybean *RAV* genes increased markedly (>2-fold). In particular, the expression of *GmRAV-01*, *GmRAV-03*, and *GmRAV-13* reached levels that were more than 10 times higher than the control (**Figure 7C**). Therefore, soybean RAV genes may be involved in ABA signal transduction pathways in soybean.

### Overexpression of *GmRAV-03* Resulted in the Transgenic Plants Insensitive to Exogenous ABA

**FIGURE 8:**
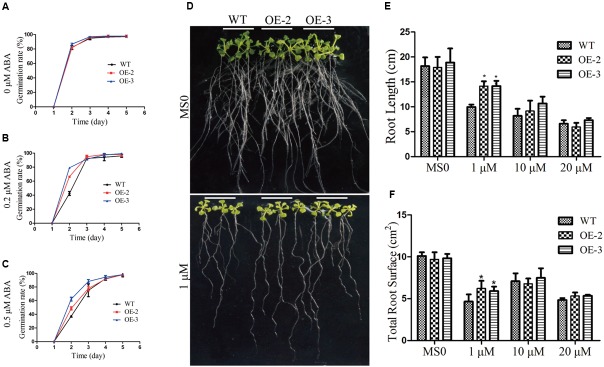
Responses of wild type and transgenic plants to ABA treatment. Seed germination assay. **(A)** Statistical analysis of seed germination on 1/2 MS medium (about 100 seeds for each line). **(B)** Statistical analysis of seed germination on 1/2 MS medium with 0.2 μM ABA. **(C)** Statistical analysis of seed germination on 1/2 MS medium with 0.5 μM ABA. **(D)** Phenotypes of WT and transgenic seedlings under ABA treatment. Four-day-old seedlings of two transgenic lines and WT *Arabidopsis* were planted on 1/2 MS medium with or without ABA for 10 days. **(E)** Comparative root lengths of WT and transgenic lines. **(F)** Total root surface of WT and transgenic lines (*n* = 27). The data are shown as mean ± SD (*n* = 27). Independent *t*-tests demonstrated that there was significant difference (^∗^*P* < 0.05).

According to the expression analysis, *GmRAV-03* was strongly induced by NaCl and exogenous ABA. To confirm the functions of *GmRAV-03* in abiotic stress responses, two homozygous *GmRAV-03* transgenic lines were selected for further analysis. For germination assays, seeds of the transgenic lines and WT germinated on 1/2 MS medium containing various concentrations of ABA, and the germination rates of both WT and transgenic seeds were determined. When germinated on 1/2 MS medium, all lines showed similar germination rate (**Figure 8A**). However, in the presence of exogenous ABA, the germination of both WT and *GmRAV-03* overexpression transgenic seeds was distinctly inhibited, but the degree of inhibition in the WT was much greater than that of transgenic seeds. With 0.2 μM ABA treatment, nearly 62–70% of the transgenic seeds germinated within 2 days, while only 40% of WT seeds germinated. The ultimate germination rate of *GmRAV-03* transgenic seeds was a litter higher than that of WT (**Figure 8B**). With 0.5 μM ABA treatment, the results were similar to that with 0.2 μM ABA treatment (**Figure 8C** and Supplementary Table S5).

**FIGURE 9:**
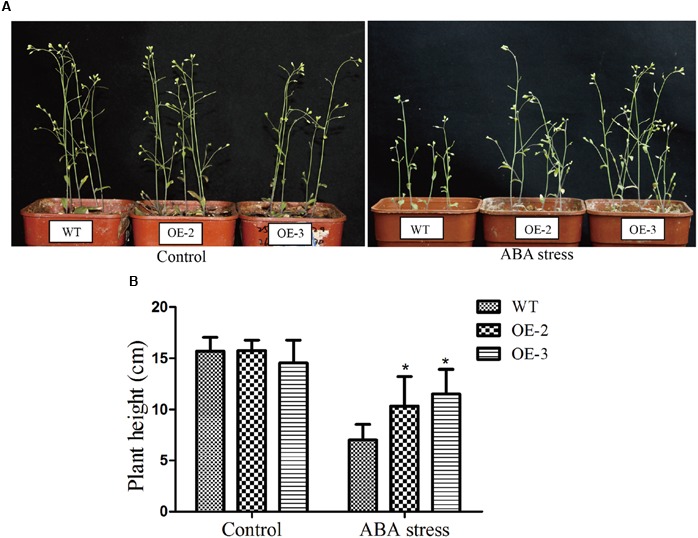
Phenotype assay of the *GmRAV-03* overexpression transgenic *Arabidopsis* grown in soil under ABA stress. **(A)** Phenotype of the *GmRAV-03* overexpression transgenic *Arabidopsis* and WT grown in soil under ABA stress**. (B)** Height of the *GmRAV-03* overexpression transgenic *Arabidopsis* and WT under ABA stress. The data are shown as mean ± SD (*n* = 45). Independent *t*-tests demonstrated that there was significant difference (^∗^*P* < 0.05).

In addition, we determined the root length and root surface of both WT and the transgenic lines (**Figure 8D**). When grown on 1/2 MS medium, all lines showed similar phenotypes. However, when grown on 1/2 MS medium containing 1 μM ABA, the root length of *GmRAV-03* overexpression transgenic seedlings were longer than that of WT (**Figure 8E**). Similarly, the root surface of *GmRAV-03* overexpression transgenic seedlings were also bigger than that of WT (**Figure 8F** and Supplementary Table S4). In seedling stage, the height of WT was slightly shorter than that of transgenic lines (**Figure 9**). These results indicated that overexpression of *GmRAV-03* in *Arabidopsis* enhances plant insensitivity to the exogenous ABA, suggesting that *GmRAV-03* may play an important role in ABA signaling during seed germination and early seedling development.

### Overexpression of *GmRAV-03* Improved *Arabidopsis* Tolerance to High Salinity

**FIGURE 10:**
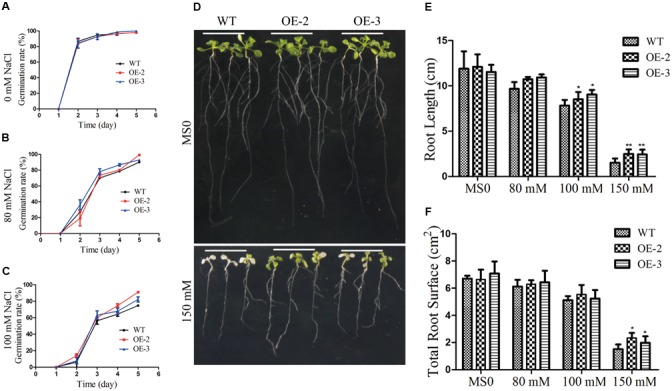
Responses of wild type and transgenic plants to NaCl treatment. **(A)** Statistical analysis of seed germination on 1/2 MS medium (about 100 seeds for each line). **(B)** Statistical analysis of seed germination on 1/2 MS medium with 80 mM NaCl. **(C)** Statistical analysis of seed germination on 1/2 MS medium with 100 mM NaCl. **(D)** Phenotypes of WT and transgenic seedlings under NaCl treatment. Four-day-old seedlings of two transgenic lines and WT *Arabidopsis* were planted on 1/2 MS medium with or without NaCl for 10 days. **(E)** Comparative root lengths of WT and transgenic lines. **(F)** Total root surface of WT and transgenic lines. The data are shown as mean ± SD (*n* = 27). Independent *t*-tests demonstrated that there was (very) significant difference (^∗^*P* < 0.05 or ^∗∗^*P* < 0.01).

To further investigate the role of *GmRAV-03* in plant growth and development under high salinity, salt tolerance of the transgenic lines was also tested. For germination arrays, seeds of the transgenic lines and WT germinated on 1/2 MS medium containing various concentrations of NaCl, and the germination rates of both WT and transgenic seeds were determined. When germinated on 1/2 MS medium, all lines showed similar germination rate (**Figure 10A**). However, in the presence of NaCl, the germination of both WT and *GmRAV-03* overexpression transgenic seeds was inhibited, but the degree of inhibition in the WT was much greater than that of transgenic seeds. The ultimate germination rate of *GmRAV-03* transgenic seeds (especially OE-2) was a litter higher than that of WT (**Figure 10B**). With 100 mM NaCl treatment, the results were similar to that with 80 mM NaCl treatment (**Figure 10C** and Supplementary Table S5).

**FIGURE 11:**
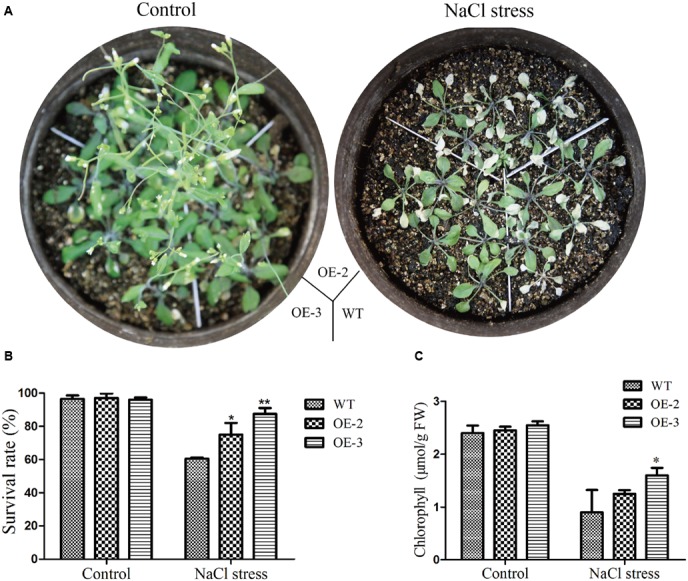
Phenotype assay of the *GmRAV-03* overexpression transgenic *Arabidopsis* grown in soil under NaCl stress. **(A)** Phenotype of the *GmRAV-03* overexpression transgenic *Arabidopsis* and WT grown in soil under NaCl stress**. (B)** Survival rate of the *GmRAV-03* overexpression transgenic *Arabidopsis* and WT under NaCl stress. **(C)** Chlorophyll content of the *GmRAV-03* overexpression transgenic *Arabidopsis* and WT under NaCl stress. 0.1 g leaves were used to extract chlorophyll. The data are shown as mean ± SD (*n* = 45). Independent *t*-tests demonstrated that there was (very) significant difference (^∗^*P* < 0.05 or ^∗∗^*P* < 0.01).

For phenotype comparison, seedlings grown on 1/2 MS medium for 5 days were transferred to 1/2 MS medium with or without NaCl. All lines showed similar phenotypes when grown on 1/2 MS medium. With 150 mM NaCl treatment for 10 days, the cotyledon of WT became yellow, even die, while the cotyledon of transgenic *Arabidopsis* still kept green (**Figure 10D**). Similarly, the root length and the root surface of *GmRAV-03* overexpression transgenic seedlings were longer than that of WT with 150 mM NaCl treatment (**Figures 10E,F** and Supplementary Table S4). Moreover, in seedling stage, survival rate, and chlorophyll content of the transgenic plants were determined and measured using WT as control, respectively. The results revealed that both survival rate and chlorophyll content of transgenic plants were higher than those of WT (**Figure 11**). Collectively, the results suggested that *GmRAV-03* may participate in plant response to high salinity, and increase the transgenic plants tolerance to salt stress.

### Overexpression of *GmRAV-03* Improved *Arabidopsis* Tolerance to Drought

**FIGURE 12:**
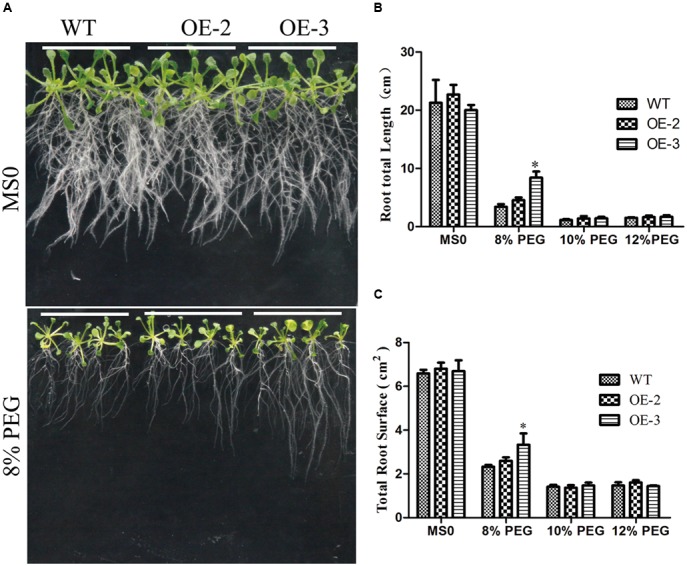
Phenotypic comparison under PEG treatment. **(A)** Phenotypic comparison. Seedlings grown on 1/2 medium for 4 days were transferred to 1/2 medium with or without PEG. **(B)** Comparative root lengths of WT and transgenic lines. **(C)** Total root surface of WT and transgenic lines. Mean values and standard errors were shown from three independent experiments (*n* = 27). Independent *t*-tests demonstrated that there was significant difference (^∗^*P* < 0.05) between the transgenic lines and WT.

**FIGURE 13:**
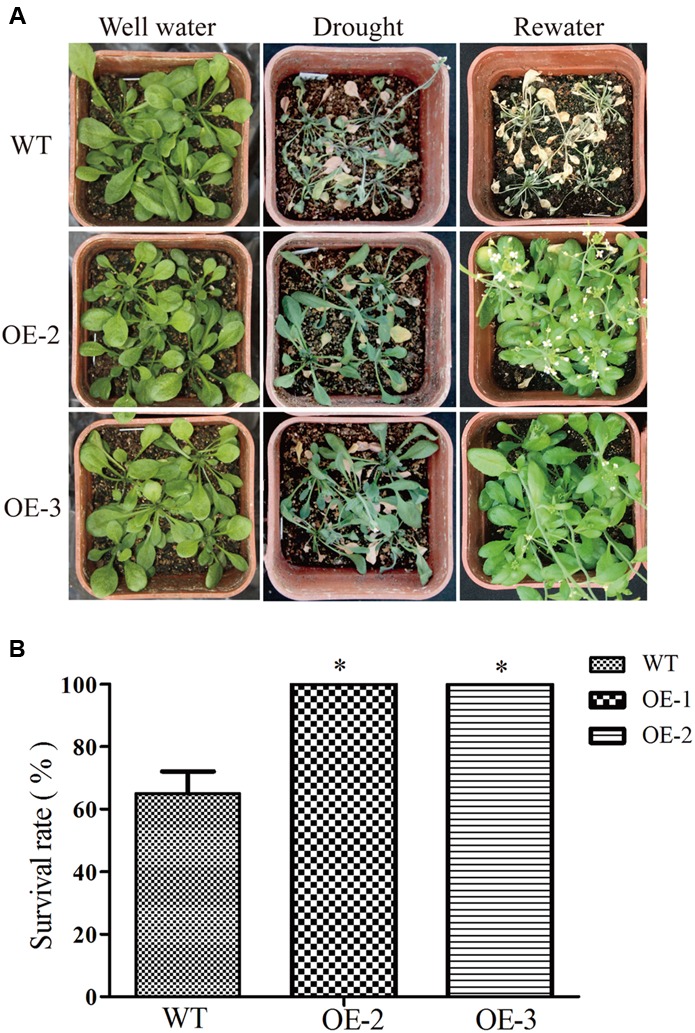
Drought tolerances in *Arabidopsis* overexpressing *GmRAV-03* and wild type. **(A)** Performance of WT and *GmRAV-03*-overexpressing lines OE2 and OE3 before and after drought stress at the seedling stage. **(B)** Survival rate of WT and *GmRAV-03*-overexpressing lines after drought stress (*n* = 3). The data are shown as mean ± SD (*n* = 45). Independent *t*-tests demonstrated that there was significant difference (^∗^*P* < 0.05).

To further investigate the role of *GmRAV-03* in drought stresses, we observed the phenotype of overexpression *GmRAV-03* transgenic *Arabidopsis* seedlings grown on medium and soil under PEG and drought treatment, respectively. In phenotype comparison, all lines showed similar phenotypes when grown on 1/2 MS medium. However, with 8% PEG for 2 weeks, the phenotypes of wild and transgenic *Arabidopsis* showed obvious difference that the root length of transgenic lines was longer than that of wild lines especially OE-3 line (**Figures 12A,B** and Supplementary Table S4). On the other hand, the total root surface of transgenic lines was also higher than that of wild lines (**Figure 12C**). And the seedlings grown in soil also showed the resistance for drought (**Figure 13A**). The survival rate of transgenic lines was higher than that of wild lines (**Figure 13B**). Given these, *GmRAV-03* may have the potential function to increase the transgenic plants tolerance to drought stresses.

## Discussion

Genome-wide analysis of the *RAV* family revealed the presence of 13 *RAV* members in soybean. There are 13 and 15 *RAV* genes in *Arabidopsis* and rice, respectively. The number of *RAV* genes in these species is relatively constant, which suggests that as a family they may have same event during plant evolution (Blanc et al., 2000) and indicates that the functions of the RAV family may be similar in these species. This is the first time that the RAV family proteins have been divided into three classes (I, II, and III) by cluster analysis in soybean. The same class probably means similar characteristic of RAV family in *Arabidopsis*, rice, and soybean. For instance, class II containing GmRAV-01, GmRAV-03, GmRAV-08, GmRAV-12, and GmRAV-13 all contain both AP2 and B3 domains. However, there was a question still existing. Why these five members showed different expression levels under various stresses? This needs to be discussed in the future and now we focused on the other aspects of RAV family and the function of the *GmRAV-03*. Research on the role of introns has made significant progress in recent years. Rose (2008) reported that introns not only function in the regulation of the gene expression (Rose, 2008), but also participate in gene evolution; this has been observed in research with mammals, nematodes, insects, fungi, and plants. Analysis of gene structures revealed that 7 of 13 *RAV* genes contain a single intron, whereas the others have no intron. Analysis of the gene chip expression results (**Figure 5**) suggests that the soybean *RAV* genes are expressed at different organs under the normal conditions. It also seems that the expression of these genes is not affected by the presence or absence of an intron. Phylogenetic analysis (**Figure 1**) showed that the number and location of *RAV* intron in the same subclass are conserved (**Figure 3**). For example, *GmRAV-02*, *GmRAV-04*, *GmRAV-07*, and *GmRAV-10*, of class III, all contain one intron.

Soybean RAV proteins as well as other transcription factors were all localized in the cell nucleus (**Figure 5**). This may indicate that RAV proteins play a role in nuclear.

*Cis-*elements in the promoters of genes play very important roles in the regulation of plant growth, development, and interaction with the environment. We analyzed the *cis*-acting elements in promoter regions of the *RAV* genes. This analysis revealed that the soybean *RAV* genes contain MYB, TC-rich repeats, and MYC elements and that the promoters of some of these genes contain ABRE, E-BOX, MBS, HSE, GT-1, and/or LTRE elements (**Table 2**). ABRE elements are known to be involved in plant responses to drought and ABA through their interaction with ABRE binding proteins (Li et al., 2012a). It was reported that the bZIP transcription factors AREB1, AREB2, and ABF3 co-regulate plant responses to osmotic stress by interacting with the ABRE elements in the *DREB2A* promoter (Kim et al., 2011). Zhu et al. (2005) mentioned that the MYB element is involved in stresses-induced drought, low temperature, salt, ABA, and GA responses (Zhu et al., 2005). LTRE elements are thought to contribute primarily to low temperature responses (Maestrini et al., 2009). Therefore, we concluded that the soybean *RAV* genes are very likely involved in plant responses to drought, salt, and ABA in plants. Our qRT-PCR results also showed the different induced mechanism in soybean RAV family for drought, salt, and ABA stresses. All of the soybean *RAV* genes were transcriptionally up-regulated by ABA and that most of the soybean *RAV* genes were down-regulated by NaCl treatment.

It was reported that soybean *Glyma10g34760*, a *RAV2-like* orthologous gene, plays a key role in cytokinin signaling and photoperiod regulation (Zhao et al., 2012). Another RAV-like transcription factor has been reported to have a role in controlling photosynthesis and senescence in soybean (Zhao et al., 2008). Two *Arabidopsis RAV* genes (*AtRAV1L and AtRAV2*) are transcriptionally down-regulated by drought and salt stress (Fu et al., 2014). However, *Brassica napus BnaRAV-1-HY15* expression is actually induced by NaCl and PEG treatments, even though it shares high identity with the *AtRAV2* of *Arabidopsis* (Zhuang et al., 2011). These studies emphasize that the functions of *RAV* genes in different species appear to be divergent.

Abscisic acid plays a crucial role in modulating plant responses to abiotic stresses. The *Arabidopsis* RAV1 transcription factor plays an important role in ABA signaling; it is a negative regulator of ABA signaling during seed germination and during early seedling development (Feng et al., 2014). In cotton, expression of *GhRAV1* is induced by ABA (Li et al., 2015), and overexpression of pepper *CARAV1* in *Arabidopsis* increases plant tolerance to drought and salt and increasing the ABA sensitivity of transgenic plants (Sohn et al., 2006). The present study revealed that all of the soybean *RAV* genes are up-regulated under ABA stress (**Figure 7C**). In contrast, most of the soybean *RAV* genes had down-regulated expression under drought and salt stress conditions (**Figures 7A,B**). These different results emphasize the functional diversity of the soybean RAVs. Although drought- and salt-stress responses in plants are often associated with ABA-signaling pathway (Zhu, 2002), Zhu reported that some osmotic stress responsive genes are induced completely dependent or independent of ABA and others are only partially ABA dependent. Perhaps, RAV gene members showed different repercussion to ABA and osomotic stresses. And this need to further be discussed and investigated in the future.

To further examine the role of RAVs, we generated the transgenic *Arabidopsis* plants overexpressing *GmRAV-03*. In this study, our results revealed that the expression of *GmRAV-03* was induced by NaCl, PEG and ABA, suggesting that *GmRAV-03* may play a role in plant response to abiotic stress and ABA signaling. Under salt, PEG, and ABA treatments, we found both seed germination, root length and root surface were increased in the transgenic lines, compared with those of WT. This may indicate that *GmRAV-03* may take part in plant response to salt and drought stresses in an ABA-independent manner.

It found that *CARAV1* enhanced the transgenic plant resistance to NaCl, but sensitive to ABA (Sohn et al., 2006). On the contrary, our data in this study revealed that *GmRAV-03* transgenic *Arabidopsis* were insensitive to both high salinity and exogenous ABA, which is consist with the previous report that the *Arabidopsis* RAV1 overexpressing lines were ABA-insensitive in terms of seed germination, root growth, and chlorophyll synthesis (Feng et al., 2014). Under salt and ABA stresses, the transgenic plants present higher germination rates, root length and root surface than those of WT, indicating that *GmRAV-03* plays an important role during seed germination and early seedling development. Considering the previous reports and the results of qRT-PCR in this study, the function of soybean RAVs may be diverse and there may be some gene-specific functions among them.

## Conclusion

Thirteen soybean *RAV* genes were identified and classified after searching the soybean genome sequence. Based on expression analyses, we conclude that the soybean RAV genes might be involved in plant responses to ABA treatment or salt, drought stresses in plants. Especially, overexpression of *GmRAV-03* could increase the transgenic lines resistance against high salt and drought and result in the transgenic plants insensitive to exogenous ABA in *Arabidopsis*. These results further indicate that soybean *RAV* genes play important roles in plant responses to abiotic and exogenous ABA stresses.

## Author Contributions

Z-SX coordinated the project, conceived and designed experiments, and edited the manuscript. S-PZ conducted the bioinformatic work, generated and analyzed data, and wrote the manuscript. W-JZ, T-FY, Y-BZ, and WZ performed experiments and analyzed data. MC, D-HM, and Y-XW provided reagents. X-HZ and S-CC contributed with valuable discussions.

## Conflict of Interest Statement

The authors declare that the research was conducted in the absence of any commercial or financial relationships that could be construed as a potential conflict of interest.

## Footnotes

1 https://www.soybase.org/soyseq/

2 http://phytozome.jgi.doe.gov/pz/portal.html

3 http://www.cbs.dtu.dk/services/Promoter/

## Supplementary Material

FIGURE S1 Relative expressions of transgenic lines and wild line (WT). qRT-PCR data were normalized using *Arabidopsis* Actin gene and shown relative to WT. X-axes showed different lines and scales of relative expression level (error bars indicate SD).

FIGURE S2 Distribution of soybean RAV genes in the genomes of soybean and *Arabidopsis*. The blue bars represent the chromosomes, and the chromosome numbers are shown on top of the bars. Note that the length of the bar has no relationship with the size of the chromosomes. The numbers on the left side of the chromosomes show the distances between the neighboring genes; the unit of distance here is the megabase (Mb). **(A)** Distribution of soybean *RAV* genes in the soybean genome. Soybean *RAV* genes are distributed on eight chromosomes. **(B)** Distribution of *Arabidopsis RAV* genes in the *Arabidopsis* genome. The *AtRAV* genes are distributed on five chromosomes.

## Abbreviations

ABA: abscisic acid
ABRE: ABA-responsive element
AREB: ABRE binding protein
DREB: DRE binding protein
GFP: green fluorescent protein
MS: Murashige and Skoog
qRT-PCR: quantitative real-time PCR
SD: short-day
WT: wild type.
