# Supplementary figures and images for: Genome-Wide Analysis of the RAV Family in Soybean and Functional Identification of GmRAV-03 Involvement in Salt and Drought Stresses and Exogenous ABA Treatment
Source: Front Plant Sci. 2017 Jun 6;8:905. doi: 10.3389/fpls.2017.00905 (PMC5459925; doi:10.3389/fpls.2017.00905)

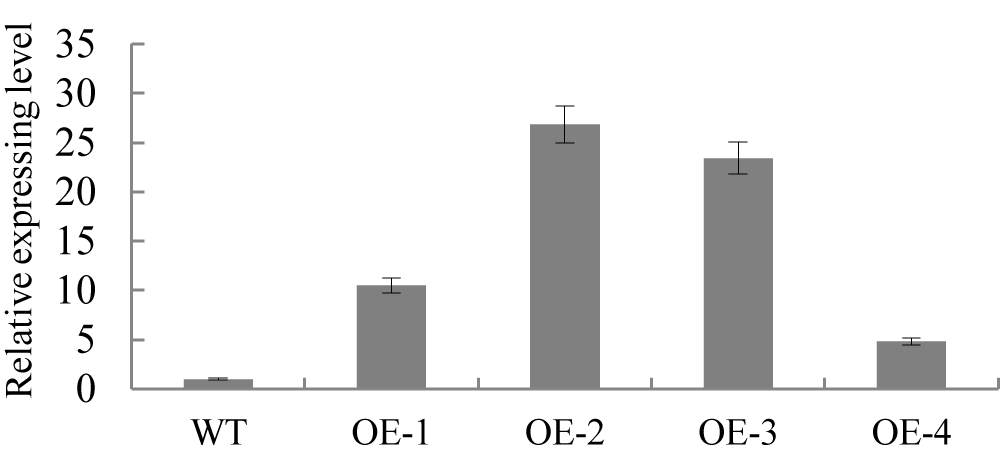

Supplement: FIGURE S1 — Relative expressions of transgenic lines and wild line (WT). qRT-PCR data were normalized using Arabidopsis Actin gene and shown relative to WT. X-axes showed different lines and scales of relative expression level (error bars indicate SD). [file Image_1.TIF]

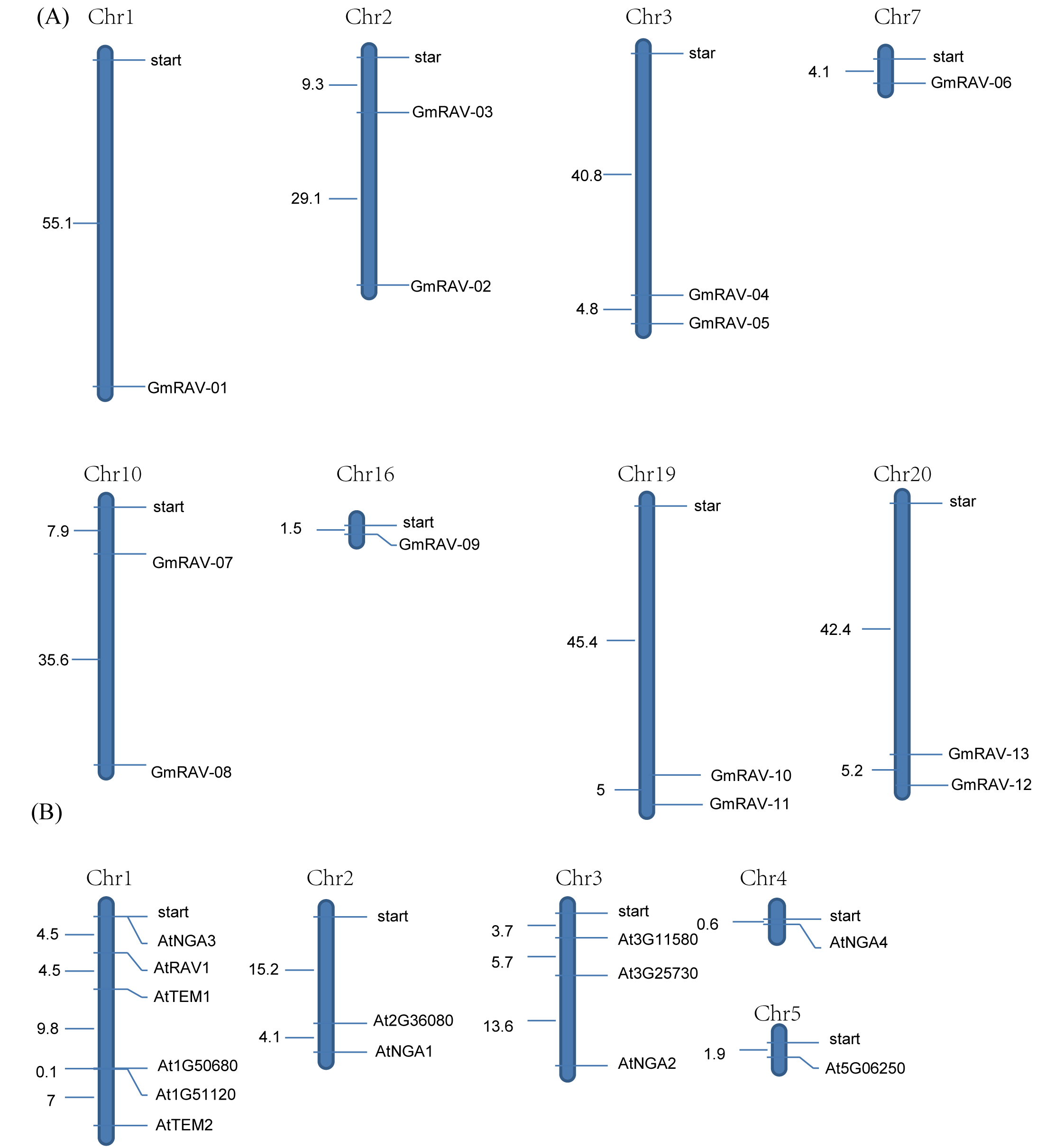

Supplement: FIGURE S2 — Distribution of soybean RAV genes in the genomes of soybean and Arabidopsis. The blue bars represent the chromosomes, and the chromosome numbers are shown on top of the bars. Note that the length of the bar has no relationship with the size of the chromosomes. The numbers on the left side of the chromosomes show the distances between the neighboring genes; the unit of distance here is the megabase (Mb). (A) Distribution of soybean RAV genes in the soybean genome. Soybean RAV genes are distributed on eight chromosomes. (B) Distribution of Arabidopsis RAV genes in the Arabidopsis genome. The AtRAV genes are distributed on five chromosomes. [file Image_2.TIF]

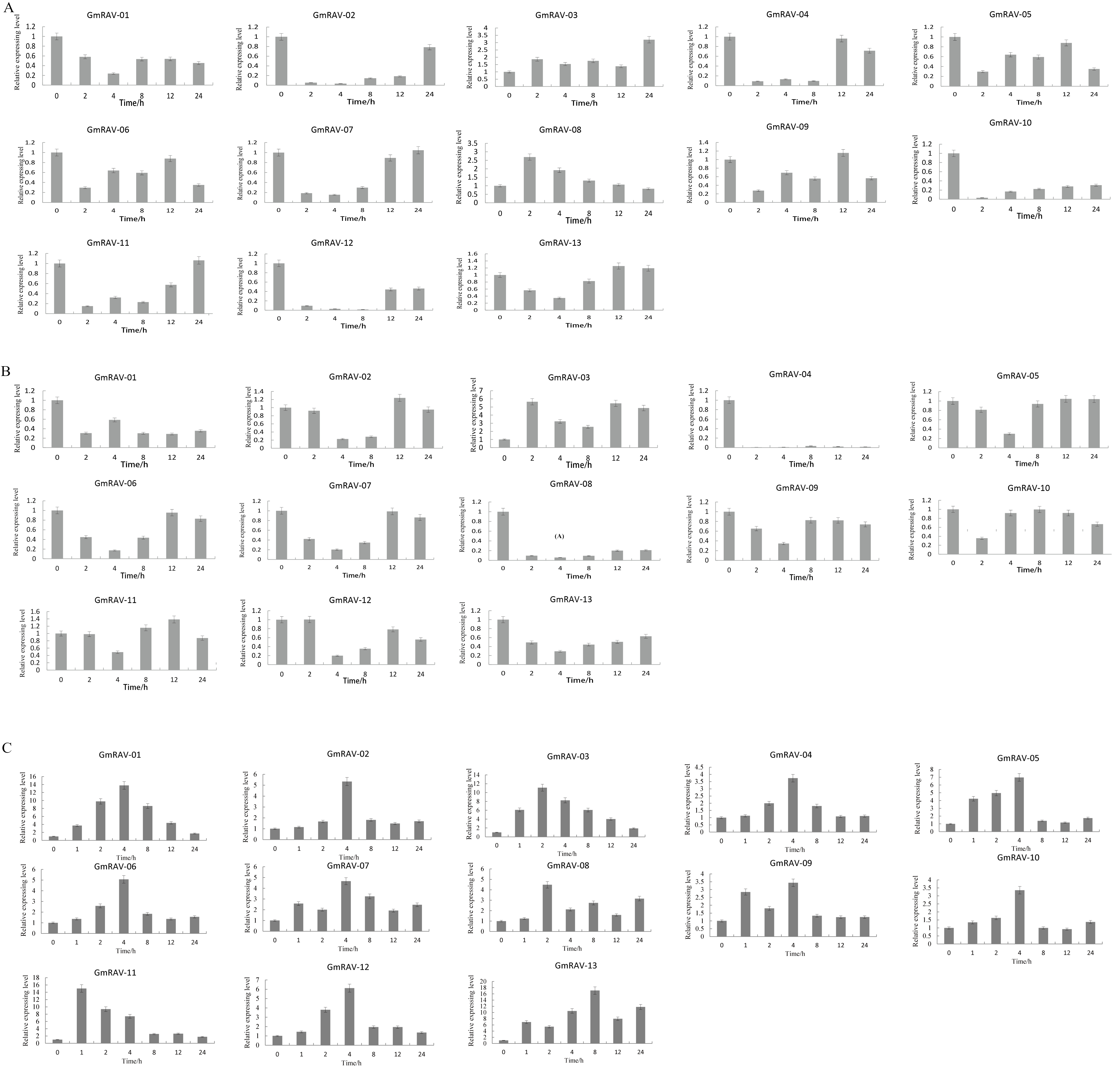

Supplement: Supplementary file 3 [file Figure_7.jpg]
