# Supplementary material for: Genome-Wide Analysis of the RAV Family in Soybean and Functional Identification of GmRAV-03 Involvement in Salt and Drought Stresses and Exogenous ABA Treatment
Source: Front Plant Sci. 2017 Jun 6;8:905. doi: 10.3389/fpls.2017.00905 (PMC5459925; doi:10.3389/fpls.2017.00905)
Supplement: Supplementary file 4 [file Table_1.DOC]

**Table S1** Primer sequences used in this study

| **Primer Name** | **Primer Sequence(5’-3’)** |
| --- | --- |
| **For qRT-PCR** | |
| Glyma01g22260F | TGACGAGCTGGAACAAAGCA |
| Glyma01g22260R | TTCAGTTTCCCAACGTCGCT |
| Glyma02g11060F | TTCAACGAGGAAGACGAGGC |
| Glyma02g11060R | CATGTCGACGATCTCGGGTT |
| Glyma02g36090F | CTACGTGCTCACCAAAGGGT |
| Glyma02g36090R | CCCATCACCACCACTCTTCC |
| Glyma03g35700F | GACGCAAGAGGAGGAGAAGG |
| Glyma03g35700R | AAGAGTAACGGAAGCGCCAA |
| Glyma03g42301F | AATGGGAAGGTGTGGCGATT |
| Glyma03g42301R | GCATGATCGGGCCTTCTCTT |
| Glyma07g05381F | TCATCATCTGGGTTAGGGTTG |
| Glyma07g05381R | ATTCCCTTCATCCTTGCTGCT |
| Glyma10g08871F | GTTTTGTTCGAGCGTCACCG |
| Glyma10g08871R | TATGGCGAGGGCTGATGATG |
| Glyma10g34760F | CGACGAAAGCTCGCCGTG |
| Glyma10g34760R | GAAGAACTGAACCGCGTCAC |
| Glyma16g01951F | CAGCCGTAAGCACCACTACT |
| Glyma16g01951R | TCCCCAGCCATGGATTTTGT |
| Glyma19g38340F | CGTCAAAGACAAACGCCTCC |
| Glyma19g38340R | ATGGGAAGGGGAGGTGATGA |
| Glyma19g45090F | GCGTTGGCCATCACAACAAT |
| Glyma19g45090R | AGTGGGTCTTCATGTGGCAG |
| Glyma20g32730F | TCTTCAAGTCGACCGGACTG |
| Glyma20g32730R | AGCCAACCCCAATACCATCC |
| Glyma20g39140F | CAATGGCTTATGACAGCGCC |
| Glyma20g39140R | ACGCCTTTTTGGGTTTGACG |
| **For Subcellular Localization Analysis**  16318hGFP Two enzyme sites: SalI and BamH I | |
| Glyma01g22260GF | CTTGCATGCCTGCAGGTCGACATGGATGCAATTAGTTGC |
| Glyma01g22260GR | TTGCTCACCATGGATCCCAAAGCACCAATAATCTTAGG |
| Glyma02g11060GF | CTTGCATGCCTGCAGGTCGACATGGATGCAATTAGTTGC |
| Glyma02g11060GR | TTGCTCACCATGGATCCCAAAGCACCAATAATCTTAGG |
| Glyma02g36090GF | CTTGCATGCCTGCAGGTCGAC ATGTCGACAAACCACTAC |
| Glyma02g36090GR | TTGCTCACCATGGATCC GTAGTAGTATGGTTGGTGAC |
| Glyma03g35700GF | CTTGCATGCCTGCAGGTCGAC ATGTCATCGATAAACCAC |
| Glyma03g35700GR | TTGCTCACCATGGATCC ATGGTTGTTGAGGTACCATGTG |
| Glyma03g42301GF | CTTGCATGCCTGCAGGTCGAC ATGGAGTTGATGCAACAA |
| Glyma03g42301GR | TTGCTCACCATGGATCC TGGCGATGATACTGCAAAGAG |
| Glyma07g05381GF | CTTGCATGCCTGCAGGTCGACATGGAGTTGATGCAAGAAG |
| Glyma07g05381GR | TTGCTCACCATGGATCCCTGCCGGTATTGCAAAGAAGGG |
| Glyma10g08871GF | CTTGCATGCCTGCAGGTCGAC ATGTCCATAAACCACTAC |
| Glyma10g08871GR | TTGCTCACCATGGATCC GTAGTAGTATGGTTGGTGATG |
| **Primer Name** | **Primer Sequence(5’-3’)** |
| Glyma10g34760GF | CTTGCATGCCTGCAGGTCGAC ATGGATGGAGGCTGTGTC |
| Glyma10g34760GR | TTGCTCACCATGGATCC CAAAGCTCCAATTACTTTTAAC |
| Glyma16g01951GF | CTTGCATGCCTGCAGGTCGAC ATGGAGTTGATGCAAGAA |
| Glyma16g01951GR | TTGCTCACCATGGATCC TCGCTTCCGGTATTGCAAAGAAG |
| Glyma19g38340GF | CTTGCATGCCTGCAGGTCGAC ATGTCATCGATACACCAC |
| Glyma19g38340GR | TTGCTCACCATGGATCC TGGTTGTTGAGGTAGCATGT |
| Glyma19g45090GF | CTTGCATGCCTGCAGGTCGAC ATGGAGTTGATGCAACAA |
| Glyma19g45090GR | TTGCTCACCATGGATCC CTGCCGATGATACTGCAAAGAG |
| Glyma20g32730GF | CTTGCATGCCTGCAGGTCGAC ATGGATGGAGGCAGTGTC |
| Glyma20g32730GR | TTGCTCACCATGGATCC CAAAGCTCCAATTACTTTTAAC |
| Glyma20g39140GF | CTTGCATGCCTGCAGGTCGACATGATTCAAACATACAAG |
| Glyma20g39140GR | TTGCTCACCATGGATCCGGTGATACACACACCA |
