# Supplementary material for: Genome-Wide Analysis of the RAV Family in Soybean and Functional Identification of GmRAV-03 Involvement in Salt and Drought Stresses and Exogenous ABA Treatment
Source: Front Plant Sci. 2017 Jun 6;8:905. doi: 10.3389/fpls.2017.00905 (PMC5459925; doi:10.3389/fpls.2017.00905)
Supplement: Supplementary file 5 [file Table_2.DOC]

Table S2 The basic formula of MS medium and 1/2MS medium (volume:1L).

|  | **MS medium** | **1/2MS medium** |
| --- | --- | --- |
| Murashige & Skoog | 4.33 g | 2.165 g |
| Glycine | 2 mg | 2 mg |
| VB1 | 0.5 mg | 0.5 mg |
| VB6 | 0.5 mg | 0.5 mg |
| Niacin | 0.5 mg | 0.5 mg |
| Sucrose | 20 g | 20 g |
| Agar | 20 g | 20 g |
