# Supplementary material for: Genome-Wide Analysis of the RAV Family in Soybean and Functional Identification of GmRAV-03 Involvement in Salt and Drought Stresses and Exogenous ABA Treatment
Source: Front Plant Sci. 2017 Jun 6;8:905. doi: 10.3389/fpls.2017.00905 (PMC5459925; doi:10.3389/fpls.2017.00905)
Supplement: Supplementary file 6 [file Table_3.DOC]

**Table S3. Digital expression analysis of soybean RAV genes**

| genes | young_leaf | | flower | one cm pod | pod shell 10DAF | | pod shell 14DAF | seed 10D  AF | seed 14D  AF | seed 21DAF | seed 25DAF | seed 28DAF | seed 35DAF | seed 42DAF | root | nodule | |
| --- | --- | --- | --- | --- | --- | --- | --- | --- | --- | --- | --- | --- | --- | --- | --- | --- | --- |
| Glyma01g22260 | 9 | | 19 | 3 | 7 | 6 | | 1 | 1 | 1 | 1 | 1 | 1 | 1 | 6 | | 1 |
| Glyma02g11060 | 9 | | 12 | 3 | 6 | 8 | | 1 | 1 | 1 | 0.1 | 1 | 2 | 2 | 6 | | 4 |
| Glyma02g36090 | 3 | 4 | | 3 | 4 | 5 | | 1 | 1 | 1 | 1 | 1 | 1 | 1 | 1 | | 19 |
| Glyma03g42301 | 7 | | 7 | 0.3 | 0.1 | 0.1 | | 0.1 | 0.1 | 1 | 2.8 | 0.1 | 0.1 | 0.1 | 1.9 | | 11 |
| Glyma03g35700 | 1 | | 1 | 1 | 1 | 2 | | 1 | 6 | 2 | 2 | 1 | 2 | 1 | 1 | | 89 |
| Glyma07G05381 | 7 | | 8.9 | 1 | 0.1 | 0.1 | | 0.2 | 0.2 | 0.1 | 2.8 | 0.1 | 1 | 0.1 | 1.7 | | 3 |
| Glyma10g34760 | 11 | | 11 | 4 | 5 | 6 | | 2 | 1 | 1 | 2 | 1 | 1 | 0.1 | 3 | | 2 |
| Glyma10g08871 | 1 | | 2 | 6 | 0.1 | 0.1 | | 0.1 | 0.1 | 0.1 | 0.1 | 0.1 | 0.1 | 0.1 | 1 | | 5 |
| Glyma16G01951 | 11 | | 9 | 1.8 | 1 | 1 | | 1 | 1 | 1 | 4.4 | 1 | 1 | 1 | 11 | | 4 |
| Glyma19g38340 | 1 | | 2 | 2 | 1 | 0.1 | | 1 | 3 | 2 | 1 | 1 | 1 | 1 | 7 | | 1 |
| Glyma19g45090 | 10 | | 5 | 1 | 1 | 1 | | 0.1 | 0.1 | 1 | 0.1 | 1 | 1 | 1 | 1 | | 14 |
| Glyma20g39140 | 4 | | 3 | 0.1 | 1 | 1 | | 0.1 | 0.1 | 0.1 | 0.1 | 1 | 1 | 1 | 1 | | 1 |
| Glyma20g32730 | 0.1 | | 0.1 | 0.1 | 0.1 | 0.1 | | 1 | 0.1 | 0.1 | 0.1 | 1 | 1 | 1 | 1 | | 0.1 |
