# Supplementary material for: Genome-Wide Analysis of the RAV Family in Soybean and Functional Identification of GmRAV-03 Involvement in Salt and Drought Stresses and Exogenous ABA Treatment
Source: Front Plant Sci. 2017 Jun 6;8:905. doi: 10.3389/fpls.2017.00905 (PMC5459925; doi:10.3389/fpls.2017.00905)
Supplement: Supplementary file 7 [file Table_4.DOC]

| **The data of root length (cm)** | | | | | | | | | |
| --- | --- | --- | --- | --- | --- | --- | --- | --- | --- |
|  | WT | | | OE-2 | | | OE-3 | | |
| | MS0 | | --- | | 10.5074 | 11.0758 | 14.0837 | 10.8872 | 13.6127 | 11.7636 | 10.6274 | 12.1098 | 11.8555 |
| 80 mM NaCl | 9.3148 | 9.1610 | 10.5295 | 10.4334 | 10.8554 | 10.8877 | 10.7088 | 11.3258 | 10.7637 |
| | 100 mM NaCl | | --- | | 8.4349 | 7.8438 | 7.2033 | 8.0808 | 9.4428 | 8.0485 | 9.5885 | 8.9528 | 8.6253 |
| 150 mM NaCl | 1.0209 | 1.6380 | 1.9137 | 1.9981 | 2.9892 | 2.4837 | 2.5844 | 2.8575 | 1.8422 |
|  |  |  |  |  |  |  |  |  |  |
| MS0 | 19.3496 | 18.9698 | 16.1541 | 20.3311 | 16.5355 | 16.8742 | 20.8693 | 16.8742 | - |
| 1 μM ABA | 10.3918 | 10.0312 | 9.4165 | 15.1922 | 13.3082 | 13.9825 | 15.3204 | 13.8421 | 13.3113 |
| 10 μM ABA | 9.1876 | - | 7.1802 | 9.7877 | 10.8452 | 6.8110 | 9.6882 | 11.6270 | - |
| 20 μM ABA | 6.6996 | 7.2582 | 5.8809 | 5.3630 | 6.9086 | 5.6131 | 6.8489 | 7.4836 | 7.6392 |
|  |  |  |  |  |  |  |  |  |  |
| MS0 | 31.5022 | 19.0877 | 21.9208 | 12.6026 | 23.4646 | 21.0156 | 27.0091 | 19.3377 | 22.1175 |
| 8% PEG | 3.9764 | 3.5774 | 2.1039 | 3.9686 | 4.3858 | 5.1975 | 3.2242 | 5.3323 | 8.8461 |
| 10% PEG | 1.5503 | 1.0240 | 0.8677 | 1.1528 | 2.5757 | 1.2949 | 1.0460 | 0.7788 | 2.1459 |
| 12% PEG | 1.5970 | 1.5537 | 1.2255 | 1.6952 | 1.5469 | 1.0883 | 2.4122 | 1.4930 | 2.1057 |
|  |  |  |  |  |  |  |  |  |  |
| **The data of total root surface (cm^2^)** | | | | | | | | | |
|  | WT | | | OE-2 | | | OE-3 | | |
| | MS0 | | --- | | 6.7018 | 6.4892 | 6.9210 | 7.4549 | 6.0706 | 6.3754 | 8.0899 | 6.4643 | 6.6929 |
| 80 mM NaCl | 5.5753 | 6.5532 | 6.2230 | 6.2992 | 6.0071 | 6.5913 | 5.6896 | 6.2484 | 7.3533 |
| | 100 mM NaCl | | --- | | 4.8006 | 5.2230 | 5.3467 | 4.7625 | 5.7912 | 6.0833 | 5.8547 | 5.2451 | 4.6101 |
| 150 mM NaCl | 1.8415 | 1.1590 | 1.5400 | 2.3876 | 1.9558 | 2.6797 | 2.4605 | 1.9558 | 1.5113 |
|  |  |  |  |  |  |  |  |  |  |
| MS0 | 10.6106 | 9.9186 | 9.7360 | 10.3886 | 9.9436 | 8.7376 | 10.4140 | 9.4140 | 9.6140 |
| 1 μM ABA | 3.6830 | 4.9530 | 5.3152 | 5.2324 | 6.3500 | 7.0612 | 5.3754 | 6.4516 | 5.8862 |
| 10 μM ABA | 6.2738 | 6.8326 | 8.1280 | 6.0706 | 7.0866 | 7.1976 | 8.7884 | 6.6040 | 7.0612 |
| 20 μM ABA | 4.6896 | 5.0084 |  | 5.4864 | 5.6388 | 4.8260 | 5.2578 | 5.4356 |  |
|  |  |  |  |  |  |  |  |  |  |
| MS0 | 6.2484 | 6.5372 | 6.5532 | 7.0292 | 6.2992 | 7.2644 | 7.3152 | 6.3500 | 6.7564 |
| 8% PEG | 2.3368 | 2.3876 | 2.1082 | 2.4892 | 2.4638 | 3.0226 | 2.3114 | 2.6162 | 3.4798 |
| 10% PEG | 1.5748 | 1.4478 | 1.1684 | 1.4732 | 1.6256 | 1.4732 | 1.1176 | 1.2700 | 1.7780 |
| 12% PEG | 1.2526 | 1.2098 | 1.6764 | 1.7526 | 1.8034 | 1.3462 | 1.6764 | 1.6256 | 1.4224 |

**Table S4 The data of root length and total root suface**
