# Supplementary material for: Genome-Wide Analysis of the RAV Family in Soybean and Functional Identification of GmRAV-03 Involvement in Salt and Drought Stresses and Exogenous ABA Treatment
Source: Front Plant Sci. 2017 Jun 6;8:905. doi: 10.3389/fpls.2017.00905 (PMC5459925; doi:10.3389/fpls.2017.00905)
Supplement: Supplementary file 8 [file Table_5.DOCX]

| Treatment | Time(day) | WT | | OE-2 | | OE-3 | |
| --- | --- | --- | --- | --- | --- | --- | --- |
| **ABA treatment** | | | | | | | |
| 0 μM ABA | 1 | 0.0 | 0.0 | 0.0 | 0.0 | 0.0 | 0.0 |
|  | 2 | 84.0 | 80.0 | 82.7 | 80.3 | 87.7 | 85.6 |
|  | 3 | 96.3 | 93.3 | 96.3 | 95.6 | 97.5 | 96.3 |
|  | 4 | 97.5 | 95.5 | 98.8 | 96.2 | 97.5 | 98.2 |
|  | 5 | 97.5 | 96.5 | 98.8 | 96.2 | 97.5 | 98.2 |
|  | | | | | | | |
| 0.2 μM ABA | 1 | 0.0 | 0.0 | 0.0 | 0.0 | 0.0 | 0.0 |
|  | 2 | 44.4 | 40.4 | 65.40 | 67.40 | 79.00 | 79.00 |
|  | 3 | 93.8 | 90.8 | 96.30 | 93.30 | 91.40 | 91.40 |
|  | 4 | 97.5 | 90.5 | 100.00 | 96.00 | 98.80 | 95.80 |
|  | 5 | 97.5 | 94.5 | 100.00 | 96.00 | 99.80 | 98.80 |
|  | | | | | | | |
| 0.5 μM ABA | 1 | 0.0 | 0.0 | 0.0 | 0.0 | 0.0 | 0.0 |
|  | 2 | 38.3 | 36.2 | 46.9 | 50.2 | 64.2 | 60.3 |
|  | 3 | 81.5 | 68.5 | 80.2 | 75.3 | 90.1 | 86.2 |
|  | 4 | 93.8 | 90.5 | 92.6 | 90.6 | 92.6 | 96.1 |
|  | 5 | 100.0 | 95.7 | 98.8 | 96.7 | 97.5 | 98.5 |
| **NaCl treatment** | | | | | | | |
| 0 mM NaCl | 1 | 0.0 | 0.0 | 0.0 | 0.0 | 0.0 | 0.0 |
|  | 2 | 86.4 | 86.9 | 87.7 | 79.5 | 88.9 | 79.9 |
|  | 3 | 95.1 | 94.2 | 95.1 | 89.9 | 95.1 | 89.9 |
|  | 4 | 97.5 | 95.1 | 97.5 | 95.6 | 98.8 | 98.2 |
|  | 5 | 98.0 | 98.0 | 98.0 | 97.7 | 100.0 | 99.7 |
|  | | | | | | | |
| 80 mM NaCl | 1 | 0.0 | 0.0 | 0.0 | 0.0 | 0.0 | 0.0 |
|  | 2 | 27.2 | 27.0 | 25.9 | 12.3 | 32.1 | 40.7 |
|  | 3 | 70.4 | 69.6 | 71.6 | 75.3 | 75.3 | 80.6 |
|  | 4 | 79.0 | 78.0 | 80.2 |  | 87.7 | 85.6 |
|  | 5 | 90.6 | 89.5 | 99.8 | 98.6 | 92.6 | 93.2 |
|  | | | | | | | |
| 100 mM NaCl | 1 | 0.0 | 0.0 | 0.0 | 0.0 | 0.0 | 0.0 |
|  | 2 | 0.04 | 12.3 | 12.3 | 15.6 | 10.3 | 5.2 |
|  | 3 | 59.3 | 53.27 | 60.3 | 59.6 | 66.7 | 59.3 |
|  | 4 | 66.0 | 62.5 | 76.3 | 72.8 | 70.5 | 65.3 |
|  | 5 | 74.1 | 75.30 | 91.5 | 90.5 | 84.3 | 80.0 |

**Table S5 The rawdata of germination under ABA and NaCl treatment**
